# Integrating 3D video tracking with the standard WHO tunnel assay: a proof-of-concept to support improving insecticide-treated nets for mosquito control

**DOI:** 10.1186/s13104-026-07860-0

**Published:** 2026-05-08

**Authors:** Beatrice H. Bredt, Mathurin Fatou, Aidi G. Lugenge, Dismas S. Kamande, Nathalie Liechti, Sarah J. Moore, Pie Müller

**Affiliations:** 1 Swiss Tropical and Public Health Institute (Swiss TPH), Allschwil, Switzerland; 2 University of Basel, Basel, Switzerland; 3 Institut Pasteur, Université Paris Cité, Paris, France; 4 Ifakara Health Institute, Bagamoyo, Tanzania; 5 Nelson Mandela African Institute of Science and Technology (NM-AIST), Tengeru, Tanzania

**Keywords:** Mosquito behaviour, Flight trajectory analysis, Behavioural bioassays, Host-seeking behaviour, Anopheles mosquitoes

## Abstract

The World Health Organization (WHO) tunnel test is a standardised laboratory assay used to characterise the biological availability and potency of active ingredients on the surface of an insecticide-treated bed net (ITN) against host-seeking mosquitoes. However, the assay provides only endpoint measurements—the proportions of mosquitoes killed and blood-fed – and therefore offers no insight into how mosquitoes interact with the ITN. Therefore, complementary behavioural data would be highly valuable, for example to reveal the extent to which mosquitoes engage with the net, thereby helping to explain differences in endpoint outcomes, or to determine the minimum assay duration required and thus improve throughput. For capturing mosquito behaviour in detail, automated three-dimensional (3D) video tracking offers a powerful approach. Here, we present a proof-of-concept study combining the WHO tunnel assay with Trackit3D, a versatile tracking system, in a laboratory in Tanzania where tunnel assays are routinely performed. The system successfully tracked multiple mosquitoes simultaneously as they were attracted to a rabbit, including measuring the duration of contact with the net, despite typical fluctuations in power supply and lighting conditions. The ability to obtain high-resolution trajectories within the WHO tunnel assay provides new opportunities to enhance the behavioural evaluation of ITNs and strengthens both the interpretability and utility of the assay.

**Supplementary Information:**

The online version contains supplementary material available at 10.1186/s13104-026-07860-0.

## Introduction

Mosquitoes are the most important vectors of human pathogens, transmitting a wide array of parasites and viruses that cause diseases such as malaria, filariasis, dengue, yellow fever, chikungunya and Zika. Among these, *Anopheles* mosquitoes, the vectors of malaria, are of particular global health importance, with an estimated 282 million malaria cases reported worldwide [1, 2]. A cornerstone of malaria vector control is the deployment of insecticide-treated nets (ITNs). Beyond providing personal protection against infective mosquito bites, the insecticide treatment also confers community-level protection by killing mosquitoes and thereby reducing malaria transmission. Since 2000, the mass distribution of ITNs, Indoor residual spraying and artemisinin-based combination therapy has contributed substantially to the global decline in malaria incidence, with ITNs having the greatest impact [3]. However, since 2015, progress has levelled off as coverage of vector control interventions and antimalarial treatment has stagnated, a situation further worsened by the rapid emergence and spread of pyrethroid resistance in mosquito populations [2].

To address growing pyrethroid resistance in *Anopheles* vectors, dual-active-ingredient ITNs—combining a pyrethroid with additional active ingredients such as chlorfenapyr or piperonyl butoxide—have been developed and have shown greater epidemiological impact than pyrethroid-only nets [4, 5]. However, for proactively delay resistance resurgence of currently effective interventions, the vector-control toolbox has to be continuously expanded through the development of new insecticides with modes of action distinct from those currently in use.

The development of ITNs incorporating new insecticides requires bioassays capable of characterising their modes of action, which may elicit diverse behavioural responses in mosquitoes. In the early product-development phase, the WHO tunnel test is the standard laboratory assay for assessing the biological availability and potency of active ingredients on ITNs [6]. Compared with other standard assays, such as cone assays, the tunnel test provides a more behaviourally relevant assessment because it allows mosquitoes to exhibit host-seeking behaviour and prolonged contact with the net [6, 7]. This is particularly important for evaluating pro-insecticides such as chlorfenapyr, for which cone assays are insufficient because of their slower mode of action and whose toxicity depends on metabolic activation in active mosquitoes [8].

However, the assay provides only endpoint measurements – the proportions of mosquitoes killed and blood-fed—and therefore offers no insight into how mosquitoes interact with the net. Complementary behavioural data could reveal the extent to which mosquitoes engage with the ITN, helping to explain differences in endpoint outcomes and improving the utility of the tunnel assay. This is especially relevant because insecticides can alter mosquito behaviour, for example by inducing ‘excito-repellency’ or reducing contact with treated surfaces, as has been observed for pyrethroids [9], thereby highlighting the importance of studying behavioural responses to dual-active-ingredient nets [10].

Chlorfenapyr-based ITNs have demonstrated high efficacy against pyrethroid-resistant mosquito populations and are expected to constitute an increasing proportion of distributed ITNs in the coming years. This further underscores the need for bioassays that accurately capture both their mode of action and their behavioural effects [5, 11].

Automated three-dimensional (3D) video tracking offers a powerful approach for capturing mosquito behaviour in detail [12] and has already been used to study mosquitoes interaction with ITNs in modified experimental set-ups [13–15]. It can also be applied in the field with wild mosquitoes [16, 17] and may prove particularly valuable for future pro-insecticides requiring metabolic activation. Here, we present a proof-of-concept study combining the standard WHO tunnel assay, which uses a rabbit as a bait, with Trackit3D, a versatile automated tracking system. The study was conducted in a laboratory in Tanzania where tunnel assays are routinely performed.

## Main text

### Experimental set-up

**Fig. 1:**
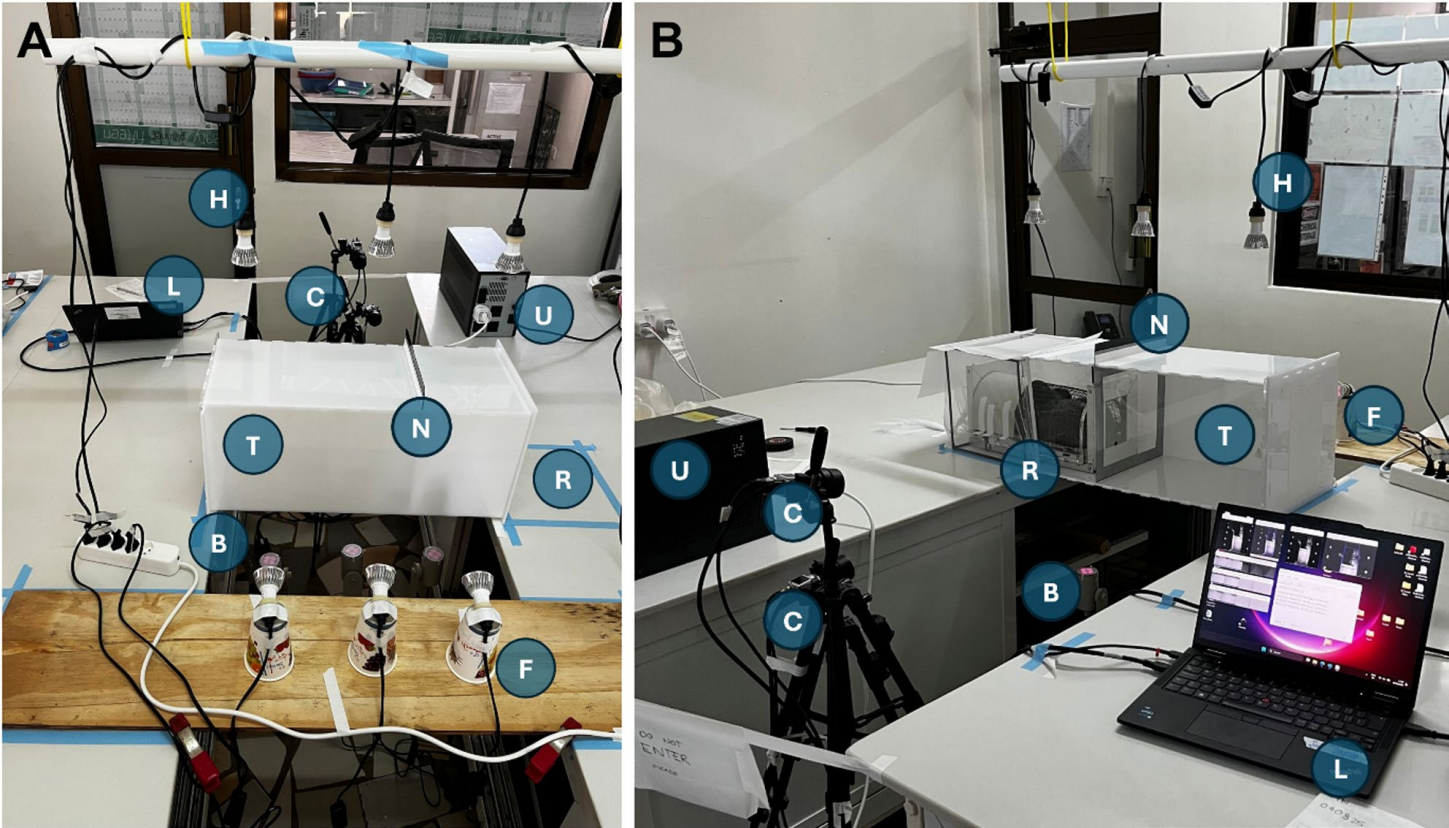
Video-tracking configuration combined with the WHO tunnel assay. The WHO tunnel consisted of three sections: a netted release chamber (not shown), a response chamber (T) and (60 cm x 25 cm x 25 cm) and a collection chamber housing the rabbit bait (R; 27 cm x 25 cm x 25 cm). A piece of netting (N) was inserted into the response chamber. The tunnel was illuminated with near-infrared light from three sides: from below (B), above (H) and from the back (F). Two cameras (C) tracked mosquito flight trajectories, powered by an uninterrupted power supply (U). The camera images were processed on a laptop (L). The camera views are shown in Supplementary Information S1, Figure S2: **A** View from the back. The response chamber containing the rabbit is not visible, but its position indicated with blue tape. **B** View from the front, with the rabbit bait (R) positioned on the left

We conducted this proof-of-concept study in a test laboratory of the Ifakara Health Institute (IHI) in Bagamoyo, Tanzania (6°26′ S, 38°53′ E). We combined a standardised WHO tunnel assay with a 3D video tracking system to record flight trajectories of mosquitoes as they negotiated the holes of an untreated net while approaching a rabbit positioned at one end of the tunnel as per the original protocol [6]. The tunnel used in this study was custom-built to facilitate video tracking and ease of transport. The side panels were laser-cut from Plexiglas and assembled by slotting them together (Fig. 1; Supplementary Information S1, Figure S1), similar to the cages described in Maire et al. [18]. This design makes the tunnel highly portable, as it can be folded into a compact form for transport. The front panel was made of transparent Perspex, while the other sides were opaque to provide a uniformly illuminated background for video tracking.

For 3D video tracking, we used Trackit3D, an automated system for recording insect flight trajectories, originally developed by Scitracks GmbH (Bertschikon, Switzerland) and now owned by Swiss TPH. The software was installed on a laptop equipped with an 11th Generation Intel^®^ Core™ i7-1185G7 processor (3 GHz) and 32 GB RAM. We mounted two acA2040-90umNIR USB 3.0 digital cameras (Basler AG, Ahrensburg, Germany), each fitted with a Fujinon DV3.4 × 3.8SA-SA1 lens (Fujifilm Holdings K.K., Tokyo, Japan) and a MidOpt BP850 near-infrared (NIR) band-pass filter (Midwest Optical Systems, Palatine, IL, USA), on tripods. We recorded flight trajectories at resolution of 4 MP and 50 fps. The system operated according to the methodology described by Fatou and Müller [13]. Potential image artefacts arising from rabbit movement were minimised by masking the rabbit cage during image processing, thereby excluding this region from tracking and object detection.

To prevent camera desynchronisation during short power outages, we connected the cameras to an APC uninterruptible power supply (Schneider Electric, West Kingston, RI, USA). All other equipment was powered directly from standard outlets, as brief interruptions did not affect performance. Tracking occasionally paused for briefly (< 1 s) during sudden NIR light loss, resulting in insufficient illumination, but resumed automatically once lighting was restored. These short interruptions were neither corrected nor manually edited, as their duration was minimal and did not affect overall trajectory reconstruction.

Illumination was provided by ten 850 nm NIR GU10 bulbs (ALLNET GmbH, Germering, Germany), fitted either in FLOTTILJ desk lamp sockets (IKEA AG, Spreitenbach, Switzerland) or Eurolite GU10 sockets with a 1.8 m cable and integrated toggle switch, allowing flexible placement around the tunnel (Fig. 1).

## Mosquito rearing

All mosquito strains were originally established from locally collected mosquitoes and since been maintained in the insectary of IHI in Bagamoyo. *Anopheles gambiae* s.s. (Ifakara strain) has been in colony since 1996 while *Anopheles arabiensis* (Kingani strain) has been colonised since 2006. *Aedes aegypti* (Kingani strain) has been maintained in colony since 2018.

Mosquitoes were maintained under standard insectary conditions in accordance with MR4 guidelines [19], at 27 ± 2 °C and 40–100% relative humidity under an approximately 12:12 h light–dark cycle. Larvae were fed Tetramin^®^ (Melle, Germany) fish food, while adults were provided with a 10% glucose solution ad libitum. For egg production, female mosquitoes were offered cattle blood via a membrane feeder or, alternatively, a human arm as a blood source.

## Experimental procedure

In a first step, we tracked, 4-6-day-old, nulliparous, non-starved female *Ae. aegypti* in an unbaited tunnel containing an untreated nine-holed piece of netting inserted in the response chamber (N in Fig. 1). This procedure allowed us to assess the initial performance of the tracking system, optimise the placement of the different elements of the set-up to ensure optimal illumination and eliminate potential reflection, and adjust the tracking parameters. We selected *Ae. aegypti* because of their diurnal activity pattern, which enabled calibration under daylight conditions. For each of the 18 calibration runs, we released ten laboratory-reared adult females into the tunnel and tracked them for 10 min under ambient conditions (28 ± 5 °C; 71 ± 10% relative humidity).

In the next step, we followed the WHO tunnel test protocol [6]. We expanded the set-up used in the first phase by adding a cage containing a rabbit (owned and bred by IHI Bagamoyo, colony maintained since 2012) as bait (Fig. 1B). To minimise movement and provide a comfortable, burrow-like environment that encouraged a natural posture, we placed the rabbit inside a smaller tunnel; however, it was not anaesthetised during the experiments. The animal’s head was shielded from light, while its hindquarters remained behind mesh, allowing mosquitoes to land and feed. In addition, we shaved the rabbit’s fur to facilitate mosquito feeding.

We then released 50 laboratory-reared, 5-8-day-old, nulliparous females of either *An. gambiae* s.s. or *An. arabiensis* into the tunnel. Mosquitoes were starved for 6–12 h prior to testing, and we tracked host-seeking behaviour for 12 h in darkness in accordance with their circadian rhythm. As in the first phase, we cross-sectionally positioned an untreated piece of netting with nine holes in the middle of the response chamber, which the mosquitoes had to navigate to reach the rabbit. In total, we conducted six replicates per strain, resulting in the exposure of 300 *An. gambiae* and 300 *An. arabiensis* females to a rabbit bait in the tunnel assay.

## Results

**Fig. 2:**
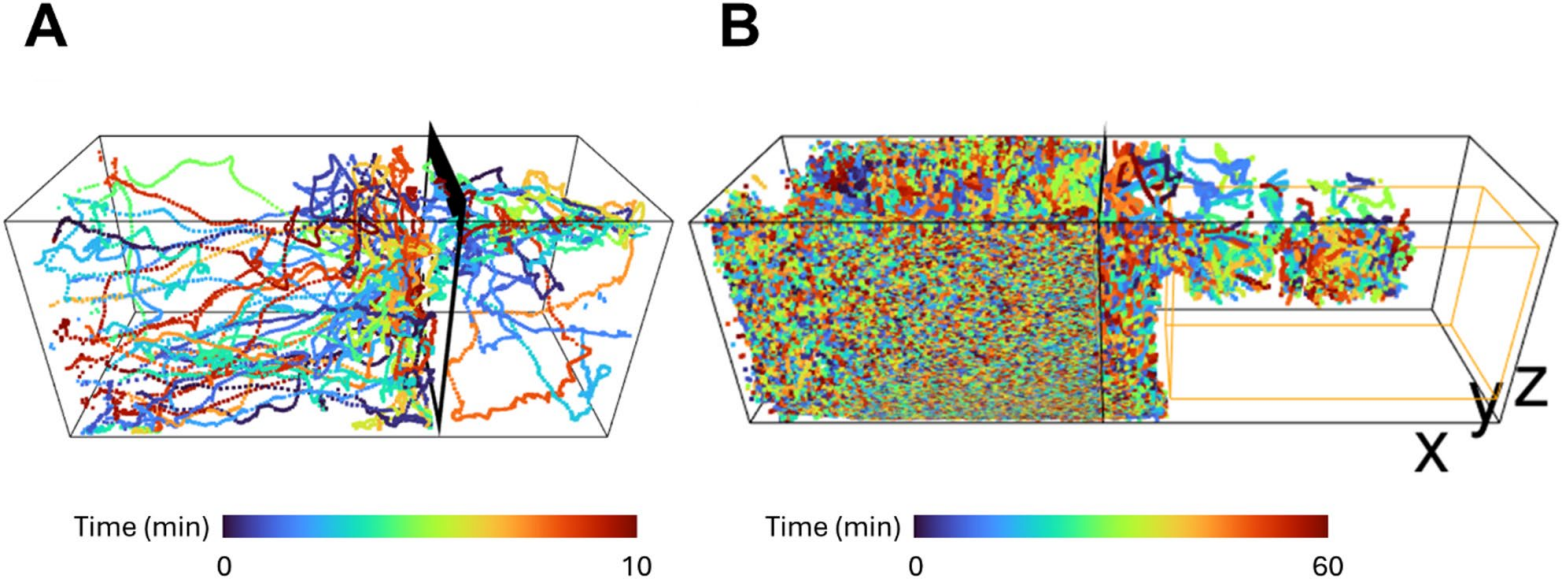
Representative mosquito flight trajectories recordedwithin the WHO tunnel assay. The colour scale indicates the timeelapsed since the start of tracking, with blue representing the beginningand dark red the end of the recording. All trajectories shown were obtainedthrough fully automated tracking and represent unedited raw data, with nomanual correction or manipulation.** A** Flight trajectories recorded from ten*Ae. aegypti* mosquitoes for 10 min in the absence of a bait, with anuntreated net in place.** B** Flight trajectories recorded from 50 An. gambiaemosquitoes for 1h (between hour 3 and 4) in the presence of a rabbit baitand an untreated net. The position of the rabbit cage is indicated by theyellow box

The video-tracking system successfully reconstructed mosquito flight trajectories within the WHO tunnel assay under both unbaited (*Aedes*) and baited (*Anopheles*) conditions (Fig. 2). Figure 2A shows representative 3D flight trajectories of ten *Ae. aegypti* females in the absence of a rabbit bait. Although no bait was present in the tunnel, the experimenters remained in the room and exhaled CO_2_ into the tunnel, eliciting host-seeking behaviour. When 50 mosquitoes were tracked over 12 h, the resulting plot became saturated with trajectories. For clarity, Fig. 2B therefore presents a 1 h subset extracted from the full 12 h recording. Of the 12 recordings, 11 were suitable for analysis; one tracking of tracking night with 50 *An. gambiae* had to be excluded because of tracking errors.

The set-up combining Trackit3D with the standardised WHO tunnel assay successfully tracked multiple mosquitoes simultaneously as they flew within the response chamber and moved through the netting holes into the collection chamber containing the rabbit. The tracking system was optimised for the region surrounding the net, where trajectory density and quality were highest. If other areas of the arena, such as the rabbit compartment, are of greater interest, the system can readily be adapted accordingly. For example, adding an additional camera on the opposite side of the arena would reduce blind spots and help mitigate the problem of mosquitoes flying behind the rabbit and becoming obscured from the front-facing cameras.

The system demonstrated stable performance despite repeated short power cuts and suboptimal lighting conditions. The flexibility and portability of both the video-tracking system and the tunnel design were critical for maintaining functionality under these conditions. To our knowledge, this is the first study to demonstrate a system capable of recording mosquito flight trajectories within a standardised WHO tunnel assay over a 12 h period.

**Fig. 3:**
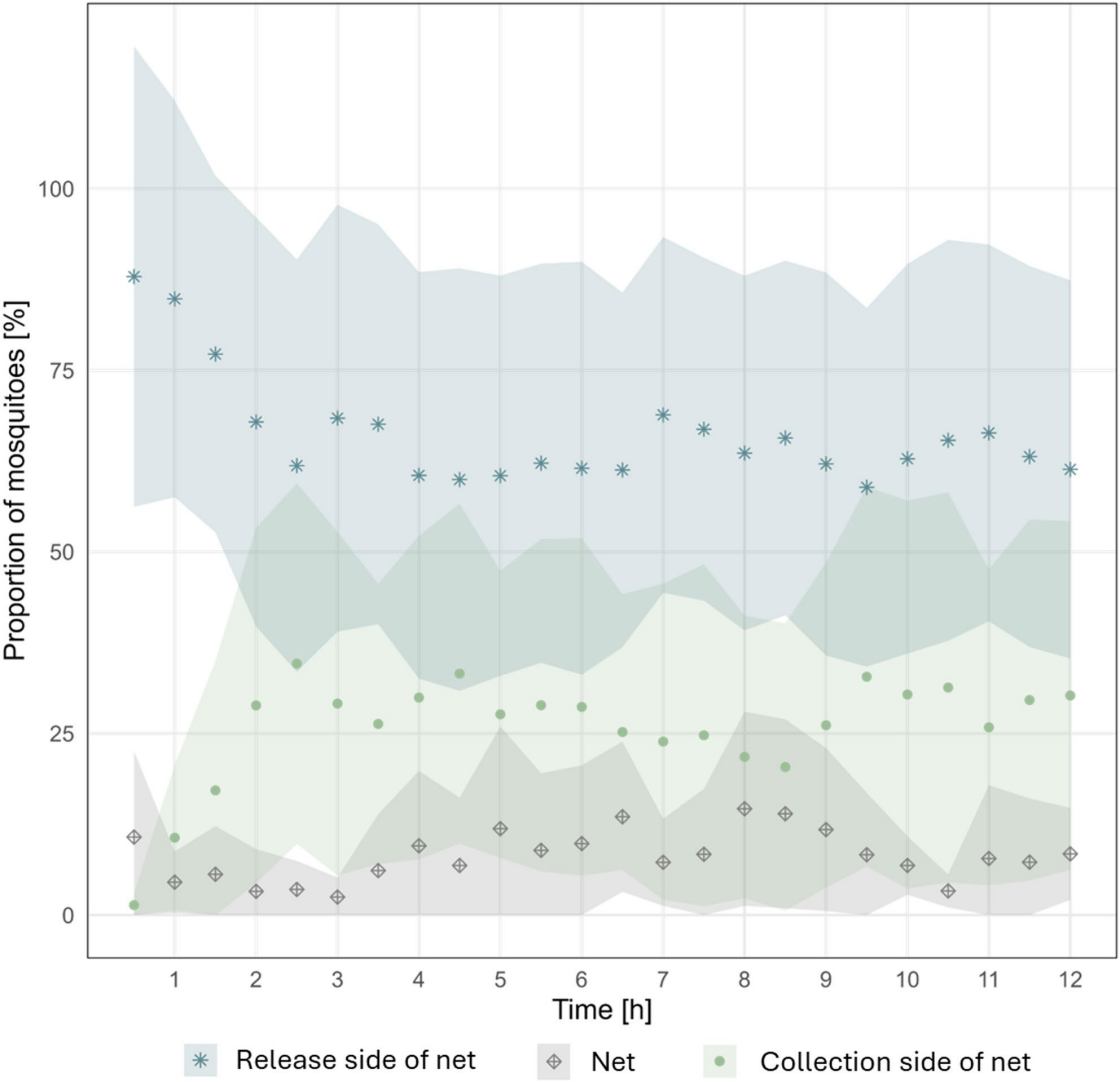
Spatial and temporal distribution of mosquitoes within the tunnel over the 12 h recording period. Following release, *Anopheles* mosquitoes were located either on the release side of the net, on the collection side containing the rabbit or at the net positioned between the two compartments. The symbols represent the mean percentage across all runs (*n* = 11), while the shaded areas indicate the 95% confidence interval around the means

The recordings enabled quantification of the time mosquitoes spent in different sections of the tunnel, particularly at the net, in the response chamber and in the collection chamber with the rabbit. They also allowed visualisation of distinct behavioural patterns, including approaching to, contact with, and resting at either the net or the bait. Figure 3 shows the spatial and temporal distribution of mosquito activity over the 12 h observation period, illustrating how long mosquitoes remained in each zone of the tunnel. On average, mosquitoes contacted with the rabbit 173,419 times, whereas approaches to and contacts with the net recorded only 12,447 times.

A previous study tracked mosquito behaviour for 10 min in a modified WHO tunnel assay using a membrane feeder as an artificial host [13]. Larger room assays to mimic experimental hut studies have also been conducted, recording mosquito interactions with ITNs over 2 h using two-dimensional video tracking while a human volunteer lay beneath the net [10]. In our study, extending the tracking period to 12 h demonstrated that mosquitoes remained active even towards the end of the assay, indicating sustained activity around untreated nets over prolonged periods. Whether assays involving ITNs should also be run for such extended durations, or whether shorter periods would suffice to increase throughput, remains an important question for future investigation. However, new active ingredients may require longer exposure periods, particularly in dual-active-ingredient nets containing pro-insecticides that depend on metabolic activation and therefore exhibit delayed effects [8].

Future studies could use this system to evaluate ITNs across a broader range of experimental settings and mosquito species. They may also investigate how mosquito behaviour varies in response to different host types and ITNs, including both natural and artificial baits. A better understanding of mosquito behaviour within the WHO tunnel assay may prove highly valuable for interpreting assay outcomes, as video tracking can bridge the gap between standardised endpoint measurements and the underlying behavioural interactions with ITNs. In particular, quantifying how mosquitoes approach, contact and interact with treated net surfaces is critical for understanding the mechanisms underlying ITN efficacy. Such insights would provide a more nuanced understanding of mosquito–net interactions and support the refinement of assays for behavioural evaluation.

## Conclusion

Our results demonstrate that Trackit3D, in combination with the foldable Plexiglas tunnel, can effectively capture mosquito behaviour within the standardised WHO tunnel assay in the presence of an animal bait such as a rabbit. The ability to generate high-resolution flight trajectories within this assay opens new opportunities to strengthen the behavioural evaluation of vector control tools and to improve the interpretability and utility of the WHO tunnel test. This proof-of-concept therefore provides a foundation for larger-scale behavioural studies and supports the development of innovative approaches for assessing vector control interventions.

## Limitations

Nonetheless, some limitations remain. The system occasionally lost track of individual mosquitoes, for example when they flew behind the rabbit or when flight paths overlapped. Reliable re-identification across repeated entry and exit events also remains a technical challenge for the long-term tracking of individual mosquitoes. Emerging neural-network-based approaches [20] may help address this by improving object recognition and overall tracking accuracy. Further advances in algorithms for object persistence, together with more efficient illumination and portable solutions will enhance future applications of the system.

## Supplementary Information

Below is the link to the electronic supplementary material.

Supplementary Material 1.

## Data Availability

The datasets generated, used and analysed during the current study, as well as the script using for data processing are available from the corresponding author on reasonable request.
