# Supplementary material for: Integrating 3D video tracking with the standard WHO tunnel assay: a proof-of-concept to support improving insecticide-treated nets for mosquito control
Source: BMC Res Notes. 2026 May 8;19:268. doi: 10.1186/s13104-026-07860-0 (PMC13321543; doi:10.1186/s13104-026-07860-0)
Supplement: Supplementary file 1 — Supplementary Material 1. [file 13104_2026_7860_MOESM1_ESM.docx]

**Supplementary Information S1**


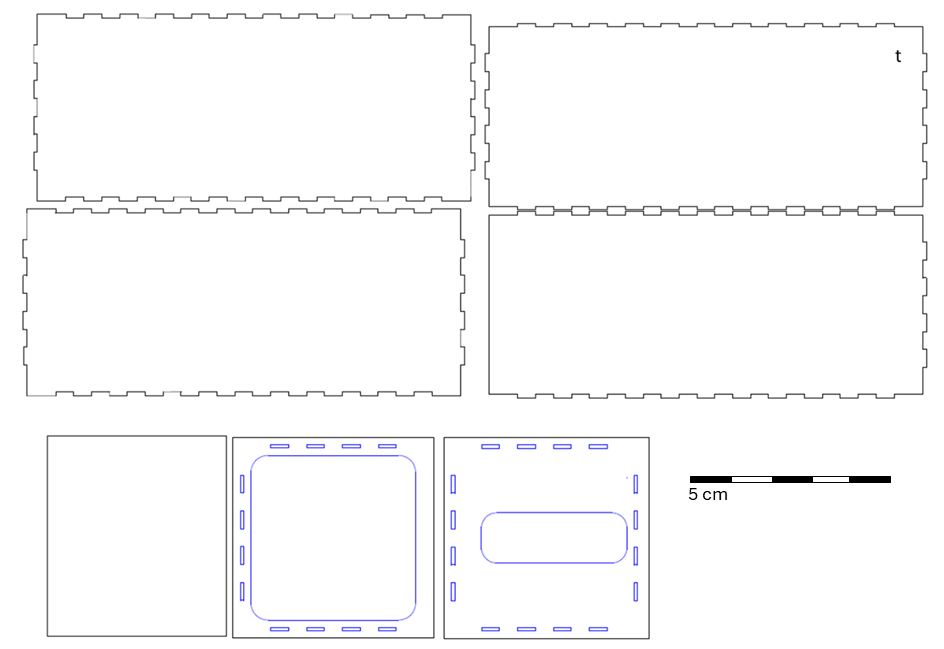


**Figure S1: CAD sketch of the dismountable Plexiglas tunnel.** Panel *t* is transparent. All other elements should be made of translucent, opaque material to create a more uniform illumination for the video tracking.


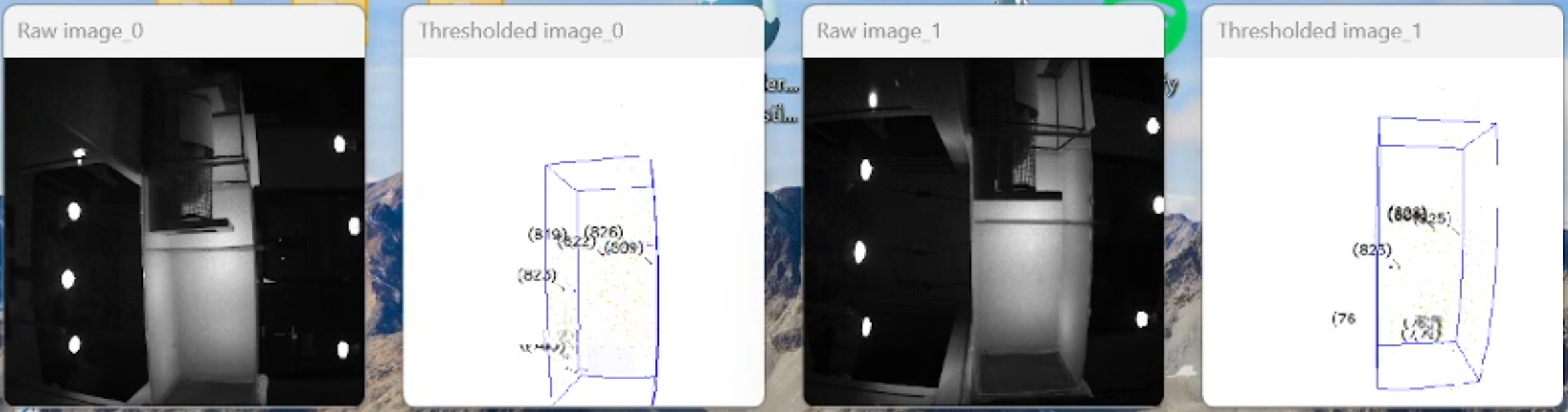


**Figure S2: Direct views from the two tracking cameras.** Images are all rotated by −90°. The ‘Raw image’ displays the original near-infrared (NIR) recording, whereas the ‘Threshold image’ illustrates the segmented tunnel space and the detected mosquito objects, labelled by object number. ‘Image 0’ corresponds to the top-mounted camera, and ‘Image 1’ to the camera positioned below the tunnel.
